# The Anterior Attentional–Intentional System in Patients with Parkinson’s Disease—A Pilot and Feasibility Study

**DOI:** 10.3390/brainsci11081013

**Published:** 2021-07-30

**Authors:** Paulina Beata Golińska, Artur Józef Sawicki, Łucja Bieleninik, Mariola Bidzan

**Affiliations:** Institute of Psychology, University of Gdańsk, 80-309 Gdańsk, Poland; artur.sawicki@ug.edu.pl (A.J.S.); lucja.bieleninik@ug.edu.pl (Ł.B.); mariola.bidzan@ug.edu.pl (M.B.)

**Keywords:** reaction time, executive functions, attention, neurodegenerative disorder

## Abstract

(1) Background: This study compared anterior attentional–intentional system performance between three groups: Parkinson’s disease (PD) patients with normal cognition (PD-NC), with mild cognitive impairment (PD-MCI), and a comparison group (CG). It also evaluated the feasibility of the recruitment and study procedures; (2) Methods: From 45 participants recruited, 39 were allocated (mean age 65.31; 43.59% men) to PD-NC, PD-MCI and CG (13 per group). To assess attention, we used three tasks from the ROtman–Baycrest Battery for Investigating Attention: Simple Reaction time (RT), Choice RT, and Prepare RT. We conducted a mixed-model analysis of variance with a 3 (groups) × 4 (tasks) design to compare reaction times; (3) Results: PD-MCI had slower reaction times than PD-NC (*p* = 0.028) and the CG (*p* = 0.052); there was no difference between PD-NC and CG. PD-MCI might perform worse on monitoring tasks than PD-NC, *Z* = −1.68, *p* = 0.092. Nearly half the volunteers from the CG and 87% of all eligible patients were enrolled in the study and completed all neuropsychological procedures; (4) Conclusions: General cognitive decline appears related to partial deficits in energization and tends to impair attentional monitoring. Furthermore, PD-NC exhibited similar reaction times to the CG. Results from the feasibility study contributed to the definitive study.

## 1. Introduction

Parkinson’s disease (PD) is the most common movement disorder and the second most common neurodegenerative disorder. The risk of PD increases with age, and it affects 1% of the population over the age of 60 globally (1–2 individuals per 1000) [1]. The main symptoms include bradykinesia, tremor, and rigidity, which are often accompanied by behavioral and cognitive difficulties [2]. Cognitive functioning may be also selectively impaired at early stages of the disease [3]. Previous studies have shown that impairments of executive functions (EF) and attention are common in patients with PD [4,5]. However, both EF and attention are heterogenous constructs, and the exact pattern of decline in PD remains unclear and debated [5]. Most models of attention emphasize the supervisory activity of the anterior attentional areas—the network of different functional parts linked to the frontal lobes [6,7]. The frontal lobes are involved in supervising cognition and behavior processes and there is clear evidence differentiating the regions of the frontal cortex and indicating its supervisory specialization [8,9]. In particular, the influential work of Stuss and Alexander, using their ROtman–Baycrest Battery to Investigate Attention (ROBBIA) methodology (based on measurement of reaction times, complemented with precise data from neuroimaging), have demonstrated that different frontal lesions lead to different supervisory difficulties with attention [6,8]. They identified that the super-medial lesion area is associated with a decrease in the process of initiating and sustaining responses (known as energization). Left dorsolateral frontal lesions result in selective difficulties with establishing stimulus–response relationships (task-setting), while right dorsolateral damage results in impairment in the process of monitoring a task over time (quality control) [6,10]. Energization, monitoring, and task-setting form together the Anterior Attentional System (AAS). The foundation for proposing the energization function resulted from neurophysiological observations—the activity of neural networks tends to become quiescent in the case of the absence of input. Moreover, according to Stuss, task-setting and monitoring processes cannot be implemented appropriately over prolonged periods without energization, which seemed to be the key AAS function following this attempt. Task-setting occurs in the initial stages of learning something new and requires establishing a criterion to respond to a specific target and organization of schemes according to the task’s requirements. Monitoring is quality control—the process responsible for checking the task performance, for example, searching for errors and implementing the potential correction [8]. However, patients with PD do not have typical cortex lesions; instead, it is more likely that they present executive and attentional problems as a consequence of disturbances in frontostriatal circuits [4,11] related to the loss of dopamine in the substantia nigra [12]. Furthermore, hypokinesis, slowness, and apathy—considered to be the cardinal symptoms of PD—are described by Stuss and Alexander as forms of energization deficits [13]. On this basis, it has been suggested that energization impairment might be the most common and characteristic disrupted attentional component in PD. Hence, the motivation for undertaking this project was to use a well-established framework for conceptualizing and testing EF/attention and to elicit distinctive characteristics of EF/attention with the ROBBIA methodology in PD patients in normal condition (PD-NC) and in PD patients with mild cognitive impairment (PD-MCI). Although there is solid evidence about reaction time impairment in individuals with PD, ROBBIA allows the interpretation of reaction times (RT) in the context of specific processes, each mediated by a different neural system in the frontal areas.

In summary, we aimed to check the feasibility of participant recruitment and assessment procedures and to evaluate the AAS—an attentional control aspect of EF and part of the Supervisory Attentional System in patients with PD in comparison to individuals without PD. To the best of our knowledge, this is the first attempt to investigate AAS in individuals with PD.

The following research questions were formulated:

(1) Feasibility of recruitment
  (1a) What percentage of eligible patients and their caregivers consent to participate in the study?
  (1b) What percentage of patients drop out after neurological examination and before neuropsychological assessment?
  (1c) Do PD patients differ in willingness to participate based on disease severity?
  (1d) What percentage of the comparison group (CG; who volunteered to participate in the study) meet the inclusion criteria?
(2) Feasibility of study procedures
  (2a) What percentage of the patients with PD are able to complete all planned neuropsychological procedures?
(3) Preliminary data
  (3a) Do patients diagnosed with PD-NC and PD-MCI demonstrate deterioration in energization compared with the CG?
  (3b) Do patients diagnosed with PD-NC and PD-MCI demonstrate deterioration in monitoring compared with the CG?
  (3c) Do patients diagnosed with PD-NC and PD-MCI demonstrate deterioration in energization compared with the CG?

We hypothesized that the functioning of AAS in PD-NC patients will be significantly less impaired than in PD-MCI patients.

## 2. Materials and Methods

### 2.1. Study Design

This pilot study was based on the cross-sectional design of a project entitled: “Characteristic frontal lobe symptoms in Parkinson’s disease”. In the pilot study we used an abbreviated version of the protocol. The protocol was approved by the Ethics Committee at the Institute of Psychology at University of Gdansk (no: 45 date of approval: 7 February 2020). Participation in the study was voluntary and every individual could refuse to participate at any point without any reason. All participants provided informed consent.

### 2.2. Participants and Setting

#### 2.2.1. Participants from Clinical Groups

The clinical group consisted of patients diagnosed with PD. The inclusion criteria were a diagnosis of idiopathic Parkinson’s disease confirmed by a neurologist according to ICD-10 criteria (code: G20) and being a right-handed native speaker of Polish. The exclusion criteria included: significant problems with vision or hearing (preventing neuropsychological tasks performance), a severe depressive episode (score in Geriatric Depression Scale > 19 points), history of other mental disorders confirmed in the interview by the neurologist (such as schizophrenia, bipolar disorder), addiction to alcohol, or other psychoactive substances, history of stroke, deep brain stimulation treatment, and epilepsy. Patients were recruited from a neurological center by a neurologist from among the PD patients being cared for there. The recruiting procedure consisted of asking each patient to voluntarily participate in the study after confirmation of the validity of their PD diagnosis. Participants from clinical groups were included without restrictions on gender, age, or educational background. Medical data, such as side of onset and PD duration, was retrieved with the patients’ consent from medical center archives.

Participants with PD were divided into the two following groups based on their cognitive status: individuals in normal condition (PD-NC) and individuals with mild cognitive impairment (PD-MCI) according to the diagnostic protocol proposed by the Movement Disorder Society [14]. Assignment to the aforementioned groups was based on the results of the neuropsychological assessment done by two independent assessors (a clinical neuropsychologist and a neurologist). Both assessments (neurological and neuropsychological) were performed in the “on” medication phase of PD patients.

#### 2.2.2. Participants from the Comparison Group

Selection to the comparison group was done by matching individuals to the patients in terms of the basic demographic variables of sex, age, and education background. The announcement about recruitment with detailed specifications was disseminated on social-media sites. The inclusion criteria were being a right-handed Polish native speaker. The exclusion criteria included: PD or suffering from neurodegenerative disorders, significant problems with vision or hearing, a severe depressive episode (score in Geriatric Depression Scale > 19 points), history of other mental diseases confirmed in the interview by the neurologist (such as schizophrenia, bipolar disorder), addiction to alcohol or other psychoactive substances, history of stroke. Furthermore, individuals from the comparison group underwent the same neuropsychological assessment. Any participant who did not receive at least 27 points on the Mini-Mental State Examination was excluded from further investigation.

### 2.3. Study Procedures

#### 2.3.1. Clinical Group

Referrals were made by a neurologist who provided an oral explanation of the proposed research project. After signing the informed consent form, individuals were referred to neurological assessments to determine the current stage of the disease (severity of disease symptoms). The neurologist also confirmed the current total daily Levodopa dose (LEDD). In the second step, patients were invited to participate in the neuropsychological assessment, which took place at the University of Gdansk. The neuropsychological procedure assumed the same order of tasks for all participants: standardized neuropsychological assessment, filling out the questionnaires, and four ROBBIA tests (in random order). All patients received oral (immediately after study) and written feedback (within two weeks) about their current cognitive functioning.

#### 2.3.2. Comparison Group

Volunteers could contact the researchers in two ways: via the registration form (on Google forms) or by phone. After the preliminary interview, including questions about inclusion and exclusion criteria, those who met the eligibility criteria were invited to the neuropsychological assessment. Participants from the comparison group underwent only the neuropsychological procedures, which were equivalent to the procedures used in the clinical groups. Similarly, the first method used in the neuropsychological assessment was Mini-Mental State Examination.

### 2.4. Data Collection and Outcome Measures

#### 2.4.1. Feasibility Data

In terms of recruitment procedure, we collected feasibility data from all individuals who were approached: how many individuals consent or refuse to participate, met the eligibility criteria, continued or withdrew from further participation, and were included in the clinical and healthy control groups. We also evaluated if there was any difference in willingness to participate in terms of disease severity.

We collected information about the use of the neuropsychological assessment among all three clinical groups—PD-NC, PD-MCI, PDD (patients with mild dementia)—during one meeting. This was a crucial point, because we wanted to be sure that all participants with PD (from each group) were able to complete all procedures. All data from the feasibility procedures are reported in terms of percentage.

#### 2.4.2. Preliminary Data

An abbreviated version of the definitive project, with a limited number of participants and measurements, was implemented to evaluate the preliminary data. In the pilot study, we used only AAS task measurement, while in the definitive project we plan to extend the procedures by including the assessment of other neuropsychological symptoms related to disruptions in frontostriatal loops, such as apathy and anosognosia. However, we need a larger sample to investigate the interplay between variables. Furthermore, in the definitive project, we will also investigate a fourth group of participants—PD patients with mild dementia (PDD)—to observe the effect of disease progression and to collect data about the caregivers’ burden and well-being. The reason for excluding patients with dementia in the pilot study was to avoid prolonging the recruitment period, which would adversely impact the project duration.

We collected data about the following demographic characteristics: age (in years), gender (male/female), education level (Primary; Lower Secondary; Upper Secondary; Bachelor’s or equivalent; Master’s or equivalent). Information about medical characteristics, such as the side of onset and PD duration, were retrieved from medical records. To assess the severity of PD, the MDS-Unified Parkinson’s Disease Rating Scale (MDS-UPDRS) and Hoehn–Yahr scale (HY scale) were used. To calculate total daily Levodopa dose, we followed the standardized Tomlison et al. formulae [15].

To assess depression, we used the full-length (30 items) Geriatric Depression Scale [16]. The Cronbach alpha coefficient of reliability in the studied groups reached 0.86. For each question. the patient could receive 0 or 1 point: higher scores indicate higher levels of depression; the cut-off point for mild depression was 10 points and was 20 points for severe depression. Cognitive functioning was assessed with the following widely used, standardized neuropsychological tests: the Mini-Mental State Examination [17], the phonemic (the letter “K” because of usage high frequency in Polish language; equivalent to the letter “F” in English) and semantic (animals) categories of the Verbal Fluency Test [18], the Digit Span from the Polish adaptation of the Wechsler Adult Intelligence Scale-Revised [19], and the Rey Complex Figure Test [20].

##### The ROtman–Baycrest Battery for Investigating Attention (ROBBIA)

This study used four subtests from the ROBBIA battery to measure reaction time (RT) in three task conditions: Simple RT (baseline reaction time measure), Choice RT, and Prepare RT [8]. At the beginning of every subtest, respondents were instructed to press the correct button in response to stimuli as fast as possible while trying to make as few errors as possible. The stimuli were one of the letters A, B, C, or D. All stimuli were black and displayed on a white background on a 21.5-inch computer monitor. Participants were asked to sit in front of the computer, approximately 50 cm from the monitor, and focus their eyes on the center of the screen. Each stimulus stayed on the screen for 7 s if a participant did not react by pressing a button. The ROBBIA subtests were designed using Psychopy, which is free software for designing reaction time tasks and recording performance data [21]. An ergonomic response pad with two large buttons was specifically manufactured to meet the participants’ dexterity needs. The tasks were preceded by a short training (6 random trials) to ensure that the instructions were well understood. The subtests used were divided according to the components of the Anterior Attentional System examined, which have been previously taken into account in other studies on AAS [8]. Detailed information about ROBBIA tasks is included in Table 1.

Simple Reaction Time (task 1). In this measurement, a capital letter “A” was presented and appeared on the screen 50 times. The participant’s task was to press button 1 (on the pad) as quickly as possible when the letter appeared on the screen. The letter disappeared when the button was pressed. Interstimulus intervals (ISI) were 3, 4, 5, 6, and 7 s long, and each interval occurred 10 times in random order. Reaction time was the time from the appearance of the stimulus on the screen to the time when the button was pressed.

Choice Reaction Time (task 2). The target object was one of the four letters A, B, C, and D; each was presented with the same frequency—each stimulus was presented with 0.25 probability over 60 trials. The ISI was the same as for Simple Reaction Time. The participant’s task was to press button 1 when a target object appeared on the screen and button 2 when other stimuli appeared on the screen.

Prepare Reaction Time (task 3 and task 4). This condition was similar to the Choice RT condition. However, in this task, there was a warning signal (WS) before each stimulus (a letter)—a star that appeared for 200 ms. The task was divided into two blocks (60 trials each, for a total of 120); in the first block, the WS appeared 1 s before the stimulus, while in the 2nd block, it appeared 3 s before the stimulus.

### 2.5. Statistical Analyses

The participants’ characteristics were examined using descriptive statistics. Percentages were used for categorical variables (followed by χ^2^ test for dichotomous variables and Kruskal–Wallis *H* test for ordinal variables), whereas means and standard deviations were used for continuous ones (followed by independent samples *t*-tests or one-way ANOVAs). Hypotheses were tested using mixed-model ANOVAs, followed by post hoc tests to examine differences between particular groups. One exception was the number of errors (to test differences in monitoring). As several errors were right-skewed, we used the non-parametric Kruskal–Wallis test, followed by pairwise comparisons done with Mann–Whitney’s *U* test. For all parametric tests, we checked assumptions of residuals’ normality (by means of standardized residuals qq plots and histograms inspection) and homogeneity of variance (using Levene’s test). The statistical significance threshold was set to *p* < 0.05. To verify the feasibility of the recruitment and assessment procedures, we used descriptive statistics as percentages.

## 3. Results

### 3.1. Study Characteristics

#### 3.1.1. Feasibility Data

**Figure 1:**
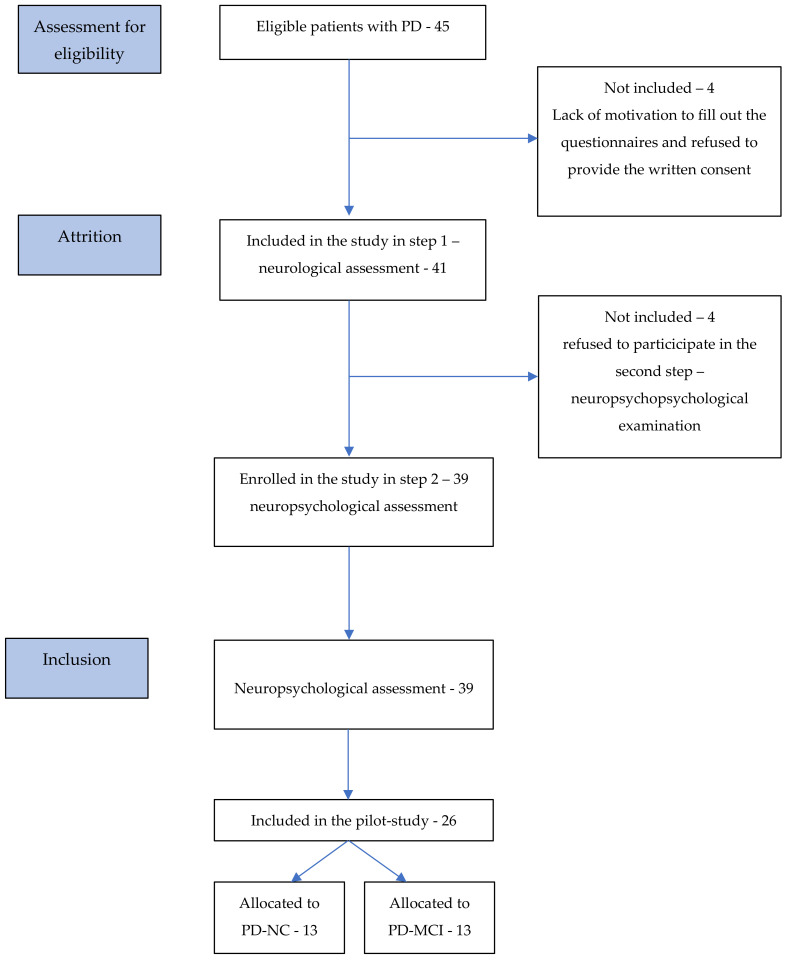
Flowchart showing how participants were recruited to the clinical group throughout the recruitment and study procedures.

From 9.02.2020 to 30.09.2020, a total of 45 participants with PD were approached, of whom 4 refused to participate in the project due to lack of motivation. From 41 (91%) potential eligible patients who were examined by the neurologist as a part of the first step of neurological assessment, 4 patients were unwilling to make a commitment and did not undergo the neuropsychological assessment (the second stage) without providing any reason for their withdrawal. Overall, 39 (87%) patients with PD were included and 26 were assigned as participants in the pilot study. A flowchart showing how participants were recruited to the clinical group is presented in Figure 1.

**Figure 2:**
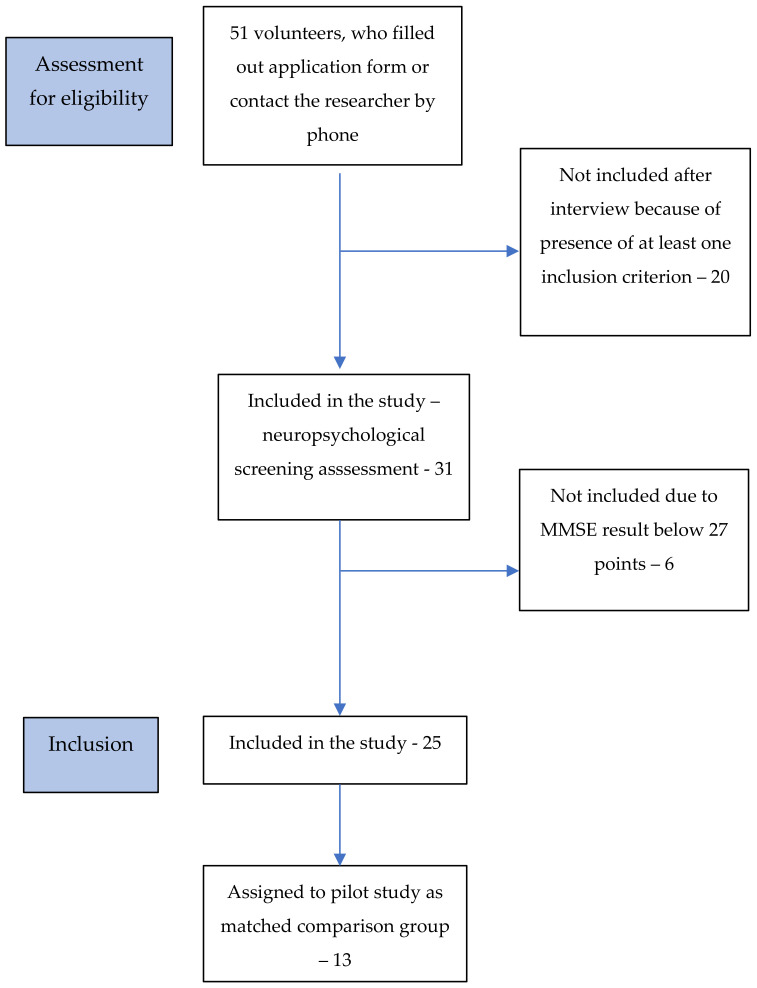
Flowchart showing the progression of the comparison group through the recruitment and study procedures.

From 1.06.2020 to 30.09.2021, a total of 51 volunteers were screened in terms of inclusion and exclusion criteria; 20 were not included in further studies due to the presence of at least one exclusion criterion. A total of 61% of volunteers qualified for the neuropsychological screening examination, of whom 6 volunteers obtained a result lower than 27 points and were excluded based on the predetermined eligibility criteria. Overall, 25 participants (49%) without PD were included in the comparison group and 13 were assigned to the study as a part of the comparison group in the pilot study. A flowchart showing how participants were recruited to the comparison group is presented in Figure 2.

#### 3.1.2. Preliminary Data

We included 39 patients in the clinical group, but after division into the three groups based on cognitive status, we obtained the following samples: PD-NC = 23; PD-MCI = 13; PDD = 3. For this reason, all 13 patients from the PD-MCI group and 13 matched patients (in terms of sociodemographic variables) from PD-NC were selected for the pilot study, to maintain equal sample sizes across the studied groups. Together, 26 patients from the clinical group were enrolled in the pilot study. A total of 13 of the 25 participants from the comparison group were included in the matched comparison group. To achieve a matched group, we first excluded individuals who significantly differed in terms of age and education and, in the second step, randomly chose 13 participants and then checked differences between groups.

In summary, the 26 individuals with PD were allocated to two groups based on their cognitive ability: 13 in the normal condition group and 13 in the mild cognitive impairment group. In addition, 13 individuals formed the comparison group. The total sample included 17 (43.59%) males of mean age 65.31 years (SD = 6.51, range: 51–79). In terms of education, the majority of those included (53.8%) had upper secondary education and 30.8% had a master’s or equivalent. There were no significant differences between groups in terms of demographic data.

More details on the demographic and clinical characteristics of the three groups are presented in Table 2. Table 3 includes additional information on results in neuropsychological tests in division into three groups. 

### 3.2. Results of Feasibility and Pilot Study

#### 3.2.1. Feasibility Data Results in Terms of Study Procedures

On average, the neurological assessment took 1 h and neuropsychological assessment about 2–3 h. We observed 100% retention rate of the participants with PD during neuropsychological procedures. Details are illustrated in Figure 1.

#### 3.2.2. Preliminary Data Results

##### Energization Results

We took reaction times across tasks as indicators of energization level. We conducted a mixed-model analysis of variance with a 3 (groups) × 4 (tasks) design to compare reaction times across the studied groups and tasks. As the group × task interaction effect was not significant, we interpreted only the main effects. There was a significant main within-subjects effect of task, *F*_(3,108)_ = 96.75, *p* < 0.001, η^2^ = 0.729. Post hoc pairwise comparisons indicated that reaction times on the Simple RT task (1) were the quickest (*p* < 0.001 in all comparisons), followed by the Prepare RT task with 1 s lag (3; *p* < 0.001 and *p* = 0.030), in comparison to the Choice RT task (2) and the Prepare RT task with 3 s lag (4), respectively. The between-subjects main effect of group did not reach the threshold of significance, *F*_(2,36)_ = 3.12, *p* = 0.056, η^2^ = 0.148. Nonetheless, given the study’s preliminary nature, relatively small sample size, and the non-trivial effect size, we decided to further analyze the data with post hoc pairwise comparisons, acknowledging an increased possibility of false-positive errors. Reaction times in the PD-MCI group were slower than in the NC group (*p* = 0.028) and marginally slower than in the comparison group (*p* = 0.052), while there was no difference between the PD-NC and comparison groups. The average reaction times in all the studied groups across the tasks are presented in Table 4.

##### Monitoring Results

To compare the groups’ functioning, we checked how they performed on a Simple RT task (task 1) and Choice RT task (task 2), taking different ISIs into account. For ease of analysis and data interpretation, we divided the ISIs into two categories: short (3 and 4 s) and long (5 and 6 s). Then, we conducted two mixed-model ANOVAs in a 3 (groups) × 2 (short ISI vs. long ISI) design, separately for the Simple RT (1) and Choice RT (2) tasks. The results indicated significant effects only for the Simple RT tasks (1). The group × ISI interaction effect was significant, *F*_(2,36)_ = 5.75, *p* = 0.007, η^2^ = 0.242; therefore, we proceeded with testing simple effects across all studied groups. Reaction times were longer when the ISI was shorter, but only in the PD-NC group (*p* < 0.001) and the PD-MCI group (*p* < 0.001), not in the comparison group (*p* = 0.364). On the other hand, intergroup comparisons across both ISI conditions showed that reaction times were slowest in the PD-MCI group, both in short ISI and long ISI conditions, all *p* < 0.035 (except the MCI vs. comparison group test in long ISI condition, where *p* = 0.061). Reaction times in all studied groups across ISI conditions are presented in Table 4.

Another indicator of monitoring system functioning that we used was the number of errors made in the Choice RT task (task 2) and both Prepare tasks (task 3 and task 4). Given the non-normal distribution of the variables (and the outliers in the PD-MCI group), we compared groups using the non-parametric Kruskal–Wallis *H* test. In general, there were no significant differences between groups on any of the tasks. However, given the study’s preliminary nature and the relatively small sample, an omnibus test (*H*_(2)_ = 5.91, *p* = 0.052) followed by pairwise *U* tests was performed for the Prepare RT with a 3 s warning interval task (4). This showed that there might be a difference between the PD-NC and PD-MCI groups, *Z* = −1.68, *p* = 0.092, but not between the others. The numbers of errors made by all studied groups across the tasks are presented in Table 5.

##### Task-Setting Results

We used the ratio of the false-positive to false-negative errors as an indicator of the functioning of the task-setting system. The predominance of false-positive errors indicates better functioning of the task-setting system. We conducted three mixed-model ANOVAs in a 3 (group) × 2 (false positive vs. false negative) design—for the Choice RT task (2) and the two Prepare RT tasks (3 and 4). The results indicate no task-setting system impairment in PD patients (all tests were not significant). The number of false-positive and false-negative errors across all examined tasks across studied groups is presented in Table 5. 

## 4. Discussion

The present study was designed to determine differences in AAS components between three groups—PD-NC, PD-MCI and CG (pilot study)—as well as to check the feasibility of the recruitment and study procedures (feasibility data).

### 4.1. Feasibility Data

The first research questions pertained to the willingness of eligible patients and their caregivers to participate in studies; we found a recruitment rate of 87%. In general, the enrollment rates were lower than expected; we therefore had to extend the duration of recruitment for 12 months to obtain the target sample size. Forty-five patients with PD were informally assessed for eligibility during the eight-month recruitment period of this study. We had initially hoped to assess twice as many participants in this period of recruitment; however, we started three weeks before the COVID-19 lockdown in Poland and were forced to suspend the research procedure for 4 months.

The pandemic likely had a negative influence on the willingness of eligible persons—both clinical and control—to participate. Taking this result into account, we must re-evaluate the timeframe for recruitment of clinical groups and extend the recruitment to different cities. We intend to start cooperation with at least two additional neurological centers to meet our current deadline of December 2021. We also observed an unequal sample size in each clinical group, which was related to differences in the willingness to participate of patients with different cognitive statuses. The persons who most frequently consent are those with no cognitive problems, followed by persons with PD-MCI. So far, we have assessed only 3 patients diagnosed with mild dementia. This is perhaps because PD-NC patients are still more active and social, so they are more motivated. Motivation is also associated with apathy, which has a high prevalence in PD. Apathy increases as the disease progresses and can be considered a relevant factor contributing to patients’ lower willingness [22]. Furthermore, another potential explanation is lack or impaired awareness of the disease. Participants with anosognosia may present lower engagement in treatment processes and participation in additional studies may not seem relevant or attractive to such patients.

The authors also observed a bias of volunteers from the comparison group with neurological diseases—a high percentage of individuals did not meet the inclusion criteria. This is probably related to the content of the announcement content and the topic of the research. It is likely that people who have neurological or psychiatric diseases may have increased interest in the field; indeed, this is the pattern we observed. The solution of using short interviews is working well and allows the authors to save time. On the other hand, all patients with PD were able to complete all neuropsychological procedures, which lasted about two hours. This confirms that the study was well designed. 

### 4.2. Preliminary Data

Despite several studies indicating attentional and executive difficulties in patients with Parkinson’s disease (PD) [5], as far as the authors are aware, there has been no research on AAS in PD. Hence, this preliminary study adds to our understanding of the selective attentional processes controlled by the frontal lobe subregions that form the AAS—energization, monitoring, and task-setting. With respect to the first research question (3a), it was found that a partial energization deficit is more related to the initiation process than to the sustaining of responses in PD-MCI. This result may suggest that the gradual system impairment in energization is related to general cognitive decline. Overall, task-setting processes seem to be preserved in PD-NC and PD-MCI. However, the tendency to deterioration in monitoring in PD-MCI requires further investigation. Overall, PD-NC present untouched AAS and no significant slowness.

Regarding the research on energization, it was found that PD-NC, PD-MCI, and the comparison group have a similar pattern of reaction times on all ROBBIA subtests: the speediest responses were observed on the Simple RT task, while the slowest responses were observed on the Choice RT task—the most demanding one. The preliminary data from the current study are consistent with results obtained in healthy individuals in other studies [8,10,22]. PD-MCI benefited from both the 1 s and 3 s warning signal on the Prepare tasks, which is not in line with our preliminary assumptions. According to Stuss and Alexander [8,10], individuals with selective damage to the super-medial region do not benefit from the 3 s warning in the Prepare task due to deficits in sustaining energized attention. However, we found that PD-MCI can maintain attention; we speculate that the general slowness might be related to impairment of the intention system caused by a delay in motor system activation, as indicated by RT slowness being independent of the difficulty of the task. At the same time, this seems to be consistent with the results of Berman’s study, indicating that the changes that occur in cognitively impaired PD are mostly driven by alterations in white matter integrity [23]. Furthermore, Yang et al. demonstrated that despite no observed impairment in performance in behavioral attentional tasks, individuals with PD-MCI may have alterations in the attention functional network and present unique cerebellum and middle frontal gyrus activation as an effective compensatory mechanism for attentional tasks [24].

This leads to the interpretation that energization decline in PD-MCI may be less specific than in patients with selective frontal lesions. Furthermore, these energization changes are also discernible in clinical practice. According to Stuss’s point of view, disrupted energization manifests in apathy, which is more widespread in cognitively impaired PD—this may support the outcomes of this study [8]. PD-NCs function as healthy comparisons, despite the loss of a significant amount of dopamine, which supports the hypothesis that levodopa tends to normalize the disrupted network topology [25]. This result is similar to that found by Zgaljardic, in which no significant changes in performance on neuropsychological tests categorized by the anterior cingulate cortex (ACC) loop were indicated in PD-NC. Nonetheless, Zgaljardic has reported that despite no interaction effect, the ACC’s functions appeared to be more severely impaired than abilities related to the orbitofrontal cortex [26]. On this basis, we hypothesize that the brain regions constituting the ACC (Brodmann’s area 24) are the same regions activated during Stuss’s Simple RT task, and these tend to be compromised in PD. The ACC circuit has projections into the basal ganglia and striatal regions. 

In response to the second research question (3b), we found that monitoring seems to be untouched: PD-NC and PD-MCI exhibit the normative decrease in RT with ISI [10]. However, there may be a tendency in general error numbers in PD-MCI, which is in line with our hypothesis. 

Furthermore, the third hypothesis was also not confirmed (3c). There were no significant between-group differences in performance on task-setting measures. This can be explained by the fact that task-setting probably remains efficient in PD, regardless of the progression of the disease. We suppose that impairment in AAS in PD may be selective and relate only to the energization process. The other hypothesis worth further investigation is task-setting performance in patients with PDD. Perhaps the deterioration in this attentional process will be observed only in advanced stages of the disease. However, this should be explored in future studies.

### 4.3. Limitations and Strengths

This is a preliminary study; therefore, the most important limitation is the small number of participants in each analyzed group—the probability of committing a type II error is high. For this reason, our interpretations of the results were cautious. A further limitation is associated with the method of measuring task-setting. It should be verified that this hypothesized component of ROBBIA is appropriate for assessing the identification of stimulus–response relationships. Despite all these limitations, we can also highlight the strengths of this study. Experimental measurement of AAS was implemented to obtain precise results corresponding to performance in each of three anterior-intentional attentional domains (energization, monitoring, and task-setting). This provides a better understanding of attentional network functioning in patients with PD and enables the interpretation of RT data in terms of single attentional processes and their neuroanatomical localizations. We also want to highlight that the methodology used—with divisions based on cognitive status—allows us to observe the progression of changes in the AAS at different disease stages. Furthermore, the feasibility assessment will help the authors to improve the procedures to be used in the definitive study.

#### Future Directions

We recommend that PD patients with dementia should be included in comparison groups to broaden our knowledge of attentional functioning patterns at different stages of the disease. These results should be also compared with classic neuropsychological test results. Moreover, the relationships between energization deficits and apathy should be analyzed.

## Figures and Tables

**Table 1:** ROBBIA subtests and tested components [3,16].

ROBBIA Subtest	Variables	Hypothesized Component Process
Simple Reaction Time (task 1)	Reaction time	Energizing
	Change in reaction time in relation to interstimulus intervals (ISI)	Monitoring
Choice Reaction Time (task 2)	Reaction time (especially in relation to the Prepare RT task)	Energizing
	Change in reaction time in relation to ISI	Monitoring
	Total number of errors	Monitoring
	Relation of false-positive to false-negative errors	Task setting
Prepare Reaction Time (tasks 3 and 4)	Reaction time (especially in relation to the Choice RT task)	Energizing
	Total number of errors	Monitoring
	Relation of false-positive to false-negative errors	Task setting

**Table 2:** Demographic information, clinical characteristics, and cognitive functioning in division into 3 groups.

	PD-NC	D-MCI	Comparison Group	Comparison Statistics
	M (SD)/*n* (%) Range			
Age	62.62 (5.94) 52–71	67.77 (5.48) 60–79	65.54 (7.37) 51–74	*F*_(2,36)_ = 2.18, *p* = 0.128
Gender (Male)	5 (38.5%)	6 (46.2%)	6 (42.2%)	χ^2^_(1)_ = 0.21, *p* = 0.901
Education level Primary Lower Secondary Upper Secondary Bachelor’s or equivalent Master’s or equivalent	0 0 7 (53.8%) 1 (7.7%) 5 (38.5%)	1 (7.7%) 2 (15.4%) 7 (53.8%) 1 (7.7%) 2 (15.4%)	0 0 7 (53.8%) 1 (7.7%) 5 (38.5%)	*H*_(2)_ = 4.32, *p* = 0.115
Geriatric Depression Scale	8.15 (7.03) 0–19	9.85 (4.79) 2–16	7.46 (4.79) 1–15	*F*_(2,36)_ = 0.62, *p* = 0.546
PD duration (months)	93.54 (70.08) 9–288	83.92 (65.20) 13–252	-	*t*_(24)_ = 0.28, *p* = 0.779
Total daily Levodopa dose	1019.62 (795.82) 280–2600	948.46 (429.36) 350–1740	-	*t*_(24)_ = 0.36, *p* = 0.720
Hoehn and Yahr stage				χ^2^_(3)_ = 8.11, *p* = 0.044
Stage 0	1 (7.7%)	0	-	
Stage I	7 (53.8%)	2 (15.4%)	-	
Stage II	5 (38.5%)	7 (53.8%)	-	
Stage III	0	4(30.8%)	-	
Side of onset				χ^2^_(2)_ = 0.90, *p* = 0.638
Left	6 (46.2%)	4 (30.8%)	-	
Right	4 (30.8%)	4 (30.8%)	-	
Bilateral	3 (23.1%)	5 (38.5%)	-	

Note: M = mean; SD = standard deviation; PD-NC = participants with Parkinson’s disease in normal cognition; PD-MCI = participants with Parkinson’s disease with mild cognitive impairment. Significant results are in bold.

**Table 3:** Additional information on cognitive functioning in division into 3 groups.

	PD-NC	PD-MCI	Comparison Group	Comparison Statistics
	M(SD)			
Mini-MentalState Examination	28.38 (1.71) 25–30	27.62 (2.14) 23–30	28.77 (1.68) 27–30	*F*_(2,36)_ = 1.64, *p* = 0.208
Phonemic fluency	15.54 (4.43) 8–22	11.92 (5.50) 5–27	15.31 (4.70) 9–26	*F*_(2,36)_ = 2.22, *p* = 0.123
Semantic fluency	21.54 (3.07) 18–28	17.85 (4.63) 10–27	22.15 (3.48) 12–25	*F*_(2,36)_ = **4.92**, *p* = **0.013**
Rey Complex Figure-copy	33.73 (2.09) 31–36	29.08 (5.51) 20–35	34.85 (2.54) 27–36	*F*_(2,36)_ = **8.87**, *p* = **0.001**
Rey Complex Figure-recall	17.62 (8.41) 2–30	11.27 (7.27) 0–24.5	16.88 (4.91) 11–30	*F*_(2,36)_ = 3.18, *p* = 0.053
Rey Complex Figure—percent recall score	52.13 (24.40) 5.88–89.60	37.48 (20.53) 0–70	48.86 (14.90) 34–91.67	*F*_(2,36)_ = 1.86, *p* = 0.170
CVLT-TOTAL ^1^	52.08 (12.52) 27–72	42.31 (9.60) 25–57	50.54 (8.33) 38–63	*F*_(2,36)_ = **3.38**, *p* = **0.045**
CVLT-TOTALREP ^2^	5.00 (7.77) 0–29	3.85 (4.08) 0–13	1.92 (1.85) 0–6	*F*_(2,36)_ = 1.17, *p* = 0.321
CVLT-LDFR ^3^	10.77 (3.39) 5–16	7.69 (3.35) 1–12	10.46 (3.15) 5–15	*F*_(2,36)_ = **3.43**, *p* = **0.043**
CVLT-LDFRREP ^4^	0.69 (1.18) 0–4	0.46 (0.52) 0–1	0.23 (0.46) 0–1	*F*_(2,36)_ = 1.12, *p* = 0.338
CVLT-RECOG ^5^	15.38 (0.96) 13–16	13.69 (2.84) 6–16	14.62 (1.98) 10–16	*F*_(2,36)_ = 2.17, *p* = 0.129
CVLT-RECOGFALS ^6^	2.85 (2.34) 0–6	4.38 (4.15) 0–14	1.31 (1.93) 0–7	*F*_(2,36)_ = **3.49**, *p* = **0.041**
Digit span ^7^	11.62 (1.76) 9–15	9.31 (2.21) 6–14	10.46 (2.54) 7–15	*F*_(2,36)_ = **3.60**, *p* = **0.038**

Note: ^1^ CVLT-TOTAL—Trials 1–5 correct responses, ^2^ CVLT-TOTALREP—Trials 1–5 repetitions, ^3^ CVLT-LDRF—Long Delay Free Recall correct responses, ^4^ CVLT-LDFRREP—Long Delay Free Recall—repetitions, ^5^ CVLT-RECOG—Recognition correct responses, ^6^ CVLT—RECOGFALS—Recognition false responses, ^7^ Digit span WAIS IV—forward and backward. Significant differences are in bold. Following post hoc tests showed that PD-MCI group scored significantly lower than two other groups in Semantic fluency, Rey Complex Figure-copy, CVLT—TOTAL, CVLT—LDFR, and Digit span. They also scored higher in CVLT—RECOGFALS^.^ There were no significant differences between PD-NC and comparison groups.

**Table 4:** Reaction times on all tasks across the studied groups.

	PD-NC	PD-MCI	Comparison Group
	M (SD)		
Simple RT	453 (54)	584 (198)	442 (95)
Choice	734 (94)	876 (272)	776 (115)
Prepare (1 s)	650 (89)	828 (350)	686 (128)
Prepare (3 s)	724 (130)	848 (247)	728 (64)
Reaction times in short and long ISI across studied groups			
	Simple RT task		
Short ISI	498 (68)	630 (224)	450 (91)
Long ISI	420 (91)	524 (175)	433 (102)
	Choice RT task		
Short ISI	728 (83)	893 (260)	781 (183)
Long ISI	744 (119)	868 (269)	778 (125)

Note: M = mean; SD = standard deviation; ISI—interstimulus intervals; PD-NC = participants with Parkinson’s disease in normal cognition; PD-MCI = participants with Parkinson’s disease with mild cognitive impairment; RT = reaction time in ms.

**Table 5:** Numbers of errors on all tasks across studied groups.

	PD-NC	PD-MCI	Comparison Group
	M (SD)		
Choice	0.85 (0.99)	1.08 (1.00)	1.31 (1.03)
Prepare (1 s)	0.92 (1.04)	5.25 (9.23)	0.85 (0.90)
Prepare (3 s)	0.69 (0.85)	1.92 (1.44)	1.00 (1.29)
Number of false-negative and false-positive errors			
	Choice RT task		
False positive	0.46 (0.52)	0.23 (0.60)	0.54 (0.52)
False negative	0.39 (0.65)	0.69 (1.03)	0.77 (0.93)
	Prepare RT task with 1 s warning interval		
False positive	0.46 (0.78)	2.00 (4.34)	0.38 (0.65)
False negative	0.46 (0.77)	2.00 (4.04)	0.46 (0.88)
	Prepare RT task with 3 s warning interval		
False positive	0.31 (0.75)	0.58 (0.51)	0.31 (0.63)
False negative	0.38 (0.65)	1.08 (1.24)	0.54 (0.66)

Note: M = mean; SD = standard deviation; PD-NC = participants with Parkinson’s disease in normal cognition; PD-MCI = participants with Parkinson’s disease with mild cognitive impairment; RT = reaction time.

## Data Availability

The research data are available at this link: https://osf.io/rp4tz/?view_only=003dd41400504203b82f7a40000a28d3, accessed on 27 July 2021.
